# How Do the Size, Charge and Shape of Nanoparticles Affect Amyloid β Aggregation on Brain Lipid Bilayer?

**DOI:** 10.1038/srep19548

**Published:** 2016-01-19

**Authors:** Yuna Kim, Ji-Hyun Park, Hyojin Lee, Jwa-Min Nam

**Affiliations:** 1 Department of Chemistry, Seoul National University, 599 Gwanak-ro, Gwanak-gu, Seoul 151-742, South Korea

## Abstract

Here, we studied the effect of the size, shape, and surface charge of Au nanoparticles (AuNPs) on amyloid beta (Aβ) aggregation on a total brain lipid-based supported lipid bilayer (brain SLB), a fluid platform that facilitates Aβ-AuNP aggregation process. We found that larger AuNPs induce large and amorphous aggregates on the brain SLB, whereas smaller AuNPs induce protofibrillar Aβ structures. Positively charged AuNPs were more strongly attracted to Aβ than negatively charged AuNPs, and the stronger interactions between AuNPs and Aβ resulted in fewer β-sheets and more random coil structures. We also compared spherical AuNPs, gold nanorods (AuNRs), and gold nanocubes (AuNCs) to study the effect of nanoparticle shape on Aβ aggregation on the brain SLB. Aβ was preferentially bound to the long axis of AuNRs and fewer fibrils were formed whereas all the facets of AuNCs interacted with Aβ to produce the fibril networks. Finally, it was revealed that different nanostructures induce different cytotoxicity on neuroblastoma cells, and, overall, smaller Aβ aggregates induce higher cytotoxicity. The results offer insight into the roles of NPs and brain SLB in Aβ aggregation on the cell membrane and can facilitate the understanding of Aβ-nanostructure co-aggregation mechanism and tuning Aβ aggregate structures.

Alzheimer’s disease (AD) is a neurodegenerative disorder; one of its pathogenic features is formation of amyloid beta (Aβ) aggregates, including amyloid plaques and neurofibrillary tangles^1^. Aβ is derived from amyloid precursor protein within the brain membrane; non-toxic Aβ can undergo structural conversion and form various toxic Aβ aggregates that are rich in β-sheet structures^2^,^3^. It is widely accepted that Aβ self-assembly is determined by its intrinsic primary sequence properties and alteration of the biological environment plays a key role in Aβ folding and accumulation^4^. Therefore, numerous studies have been conducted on the interactions between lipid membranes and Aβ and the effects of the cell membrane on Aβ aggregation^5^,^6^,^7^,^8^. Recently, we used a total brain lipid extract-based supported lipid bilayer (brain SLB) platform to study Aβ aggregation with gold nanoparticles (AuNPs)^9^. Plasmonic AuNPs were imaged for *in situ* monitoring of AuNP-Aβ aggregated structures using dark-field microscopy^9^,^10^. The results implied that the kinetics and mechanism of Aβ fibrillization can be altered by controlling the nucleation process with AuNPs. The addition of nucleation seeds were found to attenuate the lag phase of Aβ fibrillization, which inspired numerous attempts to study and control the influence of nanomaterial nucleation seeds on that process^11^,^12^,^13^,^14^. Researchers have also investigated the interaction between Aβ and nanoparticles, using engineered nanoparticles for controlling Aβ aggregation and curing amyloid-related diseases^15^. However, how the characteristics of nanoparticles, such as particle size, shape, and surface charge, affect the complicated interactions between nanoparticles, Aβ, and the brain SLB have not yet been systematically investigated. This understanding could greatly increase our knowledge of Aβ aggregation in the presence of nanoparticles and facilitate nanoparticle and lipid-based applications in diagnosing and curing Alzheimer’s disease and other protein aggregation-related diseases.

## Results

**Figure 1:**
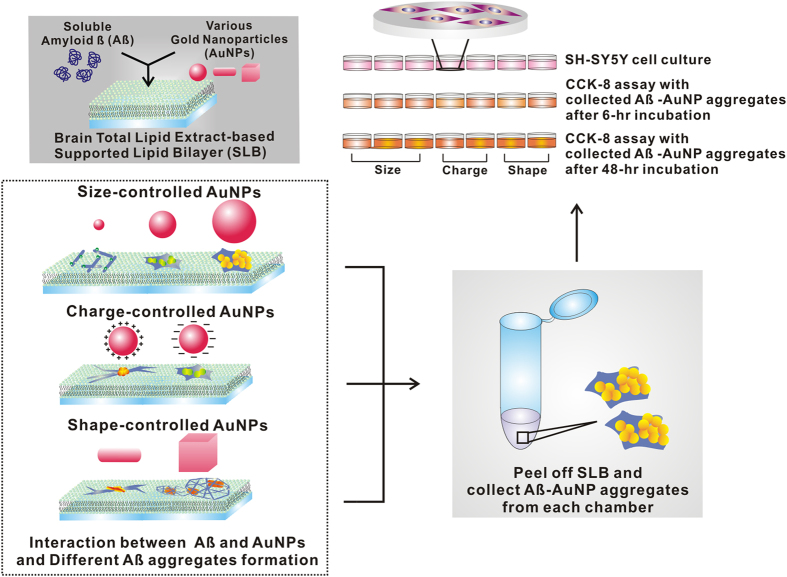
Schematic illustration of the formation of Aβ and gold nanoparticle (AuNP) co-aggregates on the total brain lipid-based supported lipid bilayer and cell viability assay with various Aβ aggregates. Depending on the size, charge, and shape of AuNPs, different Aβ aggregate structures can be formed.

We studied the effect of changes in nanoparticle size, shape, and surface charge on Aβ aggregation on a brain SLB, using AuNPs with the identical total surface area to exclude the effect of particle surface area (Fig. 1). The dark-field microscopy, Raman spectroscopy, circular dichroism spectroscopy (CD), and transmission electron microscopy (TEM) data were used as analytical tools in this study.

### Aβ incubation on the brain SLB

The SLB was prepared with 100 mol% brain total lipid extract (Avanti, Alabaster, AL, USA) including neutral and anionic lipids. Aβ can bind to anionic lipids via electrostatic interactions, which could trigger Aβ accumulation on lipid membranes^16^. The peptides adsorbed on a 2D brain SLB facilitate increase in the local peptide concentration to induce efficient peptide aggregation (Supplementary Fig. S1). This phenomenon, referred to as macromolecular crowding, favors peptide self-association as a thermodynamic and kinetic consequence^17^,^18^.

### Imaging of Aβ aggregates incubated with various sizes of AuNPs on brain SLB

**Figure 2:**
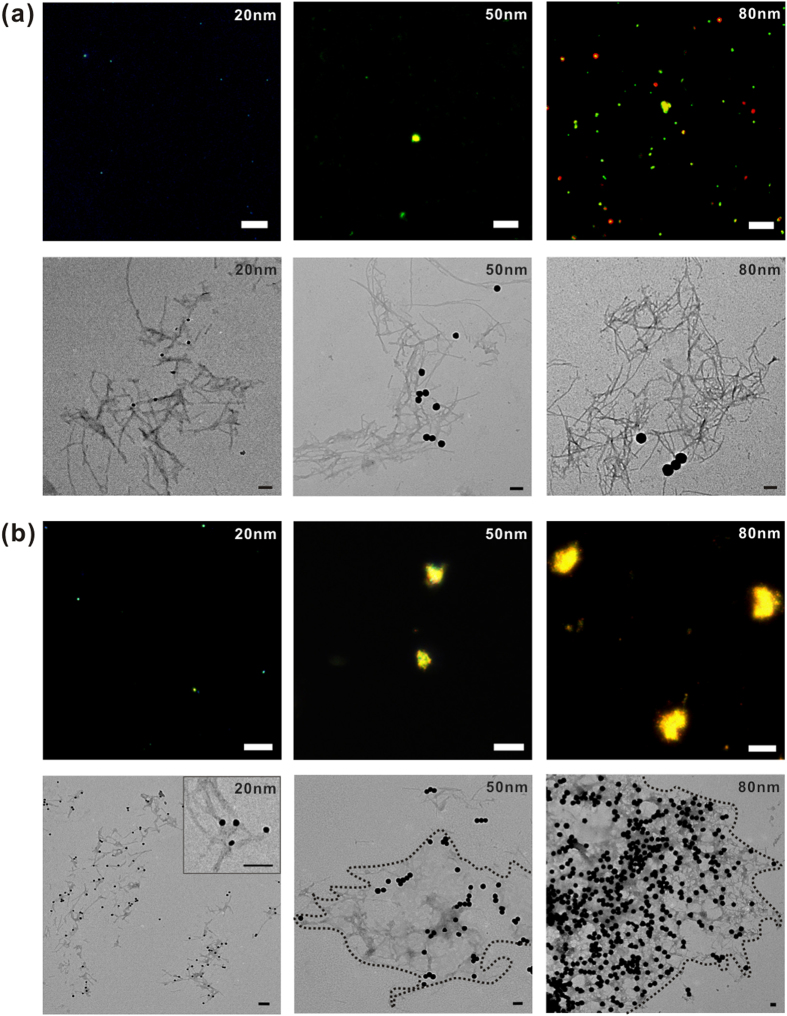
The dark-field and TEM images of Aβ aggregates with various sizes of AuNPs [20-nm AuNPs (left), 50-nm AuNPs (middle), and 80-nm AuNPs (right)] on the brain SLB. The images were obtained after the co-incubation of Aβ and AuNPs for (**a**) 6 hr and (**b**) 48 hr. The inset figure in (**b**) shows a magnified image for the 20-nm AuNP case. It should be noted that the dark-field images of 20-nm AuNPs are difficult to be obtained. The scale bars in all the dark-field images are 10 μm and those in TEM images are 100 nm.

First, AuNPs of various sizes (20, 50, or 80 nm) were co-incubated with 25 μM of Aβ monomers on the brain SLB for 6  hr and 48 hr. It was previously reported that 50 pM of 50-nm AuNP solution could induce plaque-like Aβ structures^9^ .We calculated the total surface area of 50 pM of 50-nm AuNPs and adjusted the concentrations of 20-nm and 80-nm AuNPs to retain the same total surface area in each case. In other words, 312.5 pM of 20-nm AuNPs and 19.53 pM of 80-nm AuNPs were incubated with Aβ on the brain SLB. Aβ aggregates such as Aβ oligomers, spherical aggregates, protofibrils, and fibrils are typically named for their size and structure. Aβ oligomers have a height of 2–3 nm and a width of 5–25 nm, and spherical aggregates with diameters ranging from 15–35 nm have 200–400 monomers. Protofibrils have a width of 6–10 nm and a length ranging from 5–160 nm, whereas fibrils are filamentous structures with a width of ~10 nm and a length of 0.1–10 μm^2^,^19^. After 6 hr of incubation of Aβ with 20-nm, 50-nm, or 80-nm AuNPs, they mainly formed protofibrils and short fibrils on the brain SLB; under dark-field microscopy, the color of the Aβ aggregates varied with particle size (Fig. 2). When plasmonic AuNPs are brought close to each other, the plasmonically coupled AuNPs generate color changes based on plasmon resonance wavelength shifts^20^. In the case of 20-nm AuNPs, the co-aggregates appeared green in the dark-field images, suggesting that nanoparticles had not aggregated after 6 hr (Fig. 2 and Supplementary Fig. S2). This implies that 20-nm AuNPs did not accumulate, but remained dispersed inside small Aβ aggregates. This was further supported by the TEM image shown in Fig. 2a; most 20-nm AuNPs were positioned close to each other and the AuNPs had formed protofibrils. In contrast, the larger 50-nm AuNPs induced formation of much larger Aβ aggregates, accompanied by dark-field color changes from green to greenish yellow (Fig. 2a). In the case of 80-nm AuNPs, noticeably more Aβ aggregates were formed after 6 hr (Fig. 2a). When Aβ was incubated with 20-nm AuNPs for 48 hr (Fig. 2b), more protofibrils and short fibrils were observed, and a higher number of Aβ-modified nanoparticles were observed, but they remained well dispersed. For 50-nm AuNPs, small plaque-like structures were formed with more densely modified nanoparticles. In the case of 80-nm AuNPs, many particles were densely modified to Aβ aggregates, as shown in Fig. 2b and large plaque-like structures were formed, with a dark-field color change to a reddish yellow color. Our results suggest that higher nanoparticle density results in larger Aβ aggregates. Because Aβ growth can be influenced by the accumulation of Aβ peptides on solid surfaces, we measured the amount of Aβ adsorbed on each AuNP (Supplementary Fig. S3). The 20-nm, 50-nm, and 80-nm AuNPs were measured before and after 30-min co-incubation with Aβ using the dynamic light scattering (DLS) (Zetasizer, Malvern, Worcestershire, UK). The size of nanoparticles increased due to Aβ aggregations on nanoparticle surfaces, and larger particles induced more Aβ aggregation on particle surfaces (Supplementary Fig. S3a). The size increase was also found to be correlated with the amount of Aβ peptides adsorbed on nanoparticle surface (Supplementary Fig. S3d). The results indicate that the local concentration of amyloidogenic peptides plays a key role in the Aβ growth mechanism^11^. The increased local concentration of proteins at the surface of nanoparticles could enhance the probability of partially unfolded proteins coming into frequent contact, resulting in more rapid clustering of nanoparticles and proteins^21^. In addition, it has been reported that spherical particles in protein solution are likely to form clusters, owing to short-range attraction induced by the depletion effect and the weakly screened electrostatic repulsion resulting from the modest charge^22^,^23^.

### Measurement of structural changes in Aβ-AuNP co-aggregates using SERS and CD

**Figure 3:**
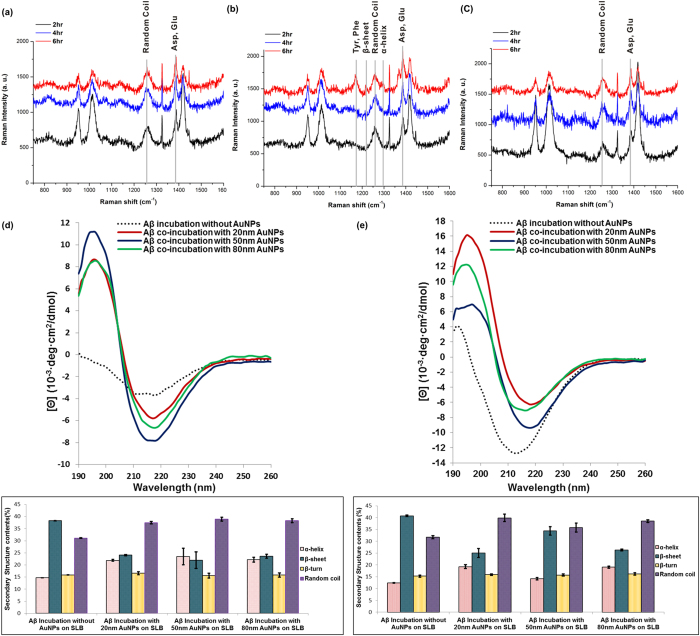
Analyses on the interactions between Aβ and AuNPs and the secondary structures of Aβ aggregates. The surface-enhanced Raman scattering (SERS) spectra of Aβ on the surfaces of (**a**) 20-nm AuNPs, (**b**) 50-nm AuNPs, and (**c**) 80-nm AuNPs with varying incubation time. Circular-dichroism (CD) measurements and secondary structure analysis after co-incubation of Aβ and AuNPs for (**d**) 6 hr and (**e**) 48 hr. The error bars were calculated with three individual samples.

We studied how Aβ secondary structures affected after incubation with AuNPs on the brain SLB using SERS and CD spectra (Fig. 3). We measured SERS signals to investigate the interactions between Aβ and AuNPs-the adsorption of molecules onto metal surfaces result in the SERS through electromagnetic field enhancements^24^,^25^,^26^. Therefore, the SERS analysis could elucidate which specific residues of Aβ are strongly bound to the AuNP surface. In the case of 20-nm and 80-nm AuNPs (Fig. 3a,c), no significant changes were observed on the surface of the AuNPs. Several peaks were assigned to random coil structures, CH_2_ symmetric rocking, CH_2_, CH_3_ deformation, the S = O of Met, and the COO¯ stretching of Asp and Glu. Interestingly, 50-nm AuNPs induced different Raman signals-after 6 hr of incubation, random coil structures as well as β-sheet and α-helix structures were clearly observed (Fig. 3b). It appeared that conformational changes in Aβ peptides, from random coils to β-sheets or α-helices, were more prevalent on the surface of 50-nm AuNPs than 20-nm and 80-nm AuNPs. These results were further compared to the CD to study changes in protein secondary structures^27^. We used 1-mm quartz cells containing the SLB, incubated under the same conditions used for the dark-field and TEM imaging experiments. After 6 hr of incubation, random coil structures were prevalent in the Aβ aggregates containing 20-nm, 50-nm, and 80-nm AuNPs; many Aβ peptides were stacked on the surface of the AuNPs (Fig. 3d). With a short incubation time, fewer folded structures contained β-sheets, even though co-aggregates with protofibrils were observed. These results are supported by a previous report, which stated that random coil structures were mainly observed following co-incubation with AuNPs^9^, resulting from structural perturbation of the surface-bound state of the protein^28^. Under these conditions, adsorbed Aβ peptides are strongly constrained, in quasi-2D, and therefore favor conversion into random coils as opposed to free Aβ monomers^29^,^30^. In the case of 50-nm AuNPs, the number of β-sheet structures increased and the number of α-helix and random coils decreased as incubation time increased. The 20-nm and 80-nm AuNPs samples formed fewer α-helices or β-sheets and more random coils, but the amount of β-sheet structures increased slightly as incubation time increased (Fig. 3e). Thus, the 20-nm AuNPs could not act as nucleation seeds within a short incubation time and were not sufficient to form entangled co-aggregates with Aβ, inducing protofibrils and short fibrils owing to the small surface area and low volume fraction of the particles. However, both the 50-nm and 80-nm AuNPs could shorten the lag phase of Aβ aggregation, and 50-nm AuNPs in particular showed the potential to increase growth of Aβ folded structures rich in β-sheets, with plaque-like structures in which Aβ and AuNPs clustered together. These plaque-like structures were similar to amyloid plaques in the AD brain, which are composed of interwoven masses of fibrils^31^. Co-incubation of 80-nm AuNPs showed that larger nanoparticles inhibit Aβ aggregation, even though the particles’ larger surface area provided more binding sites for nucleation. It should be noted that Aβ aggregates grown on the surface of 80-nm AuNPs have lower percentage of β-sheet than 50-nm AuNPs. 80-nm AuNPs with the surface-bound Aβ peptides tend to be more clustered to form large Aβ aggregates than the 50-nm AuNP case, and slight increase in both α-helix and random coil structures was observed in the CD data for 80-nm AuNPs. Moreover, based on the Raman data (Fig. 3b), the conformational changes of Aβ peptides from random coils to β-sheets or α-helices were more dominant for 50-nm AuNPs than 80-nm AuNPs. The results suggest that Aβ aggregates have more β-sheet structures in the case of 50-nm AuNPs while 80-nm AuNPs induce more alpha helix and random coil structures in Aβ aggregates, forming more amorphous peptide aggregates. The largely clustered 80-nm AuNPs induce AuNP aggregation-driven Aβ-AuNP co-aggregate structures while 50-nm AuNPs simply offer Aβ aggregation platforms and the aggregation between Aβ peptides are more prevalent in this case.

### Aβ-AuNP co-aggregates formation with differently surface-charged AuNPs

**Figure 4:**
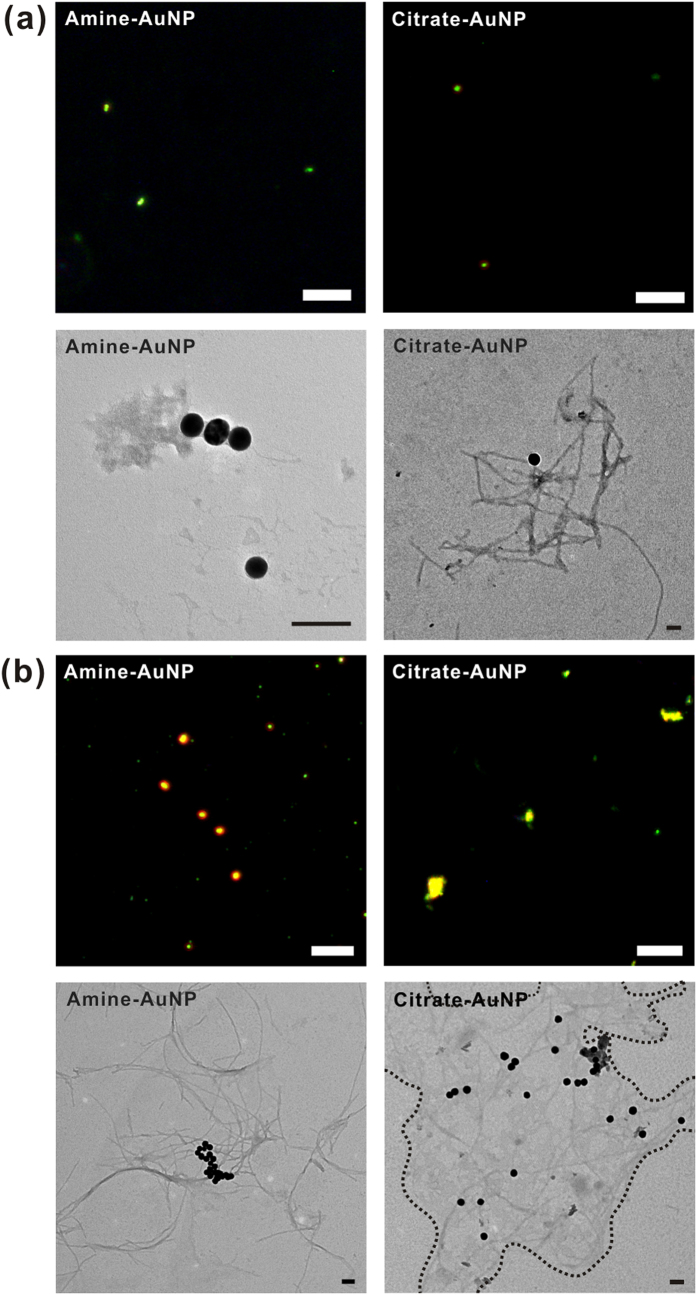
The dark-field and TEM images of Aβ-AuNPs co-aggregates with positively or negatively-charged AuNPs on the brain SLB after 6-hr and 48-hr co-incubation. Aβ and 40-nm AuNPs were co-incubated for (**a**) 6 hr and (**b**) 48 hr. The scale bars in the dark-field images are 10 μm whereas those in the TEM images are 100 nm.

It has been reported that AuNPs with modified surface charges can alter the Aβ aggregation pathway and induce differing cytotoxicity to neuroblastoma cells^32^. To investigate how surface charge influences Aβ aggregation, amine-modified AuNPs (amine-AuNPs) with positive charges were synthesized (see Supplementary Fig. S4 and Supplementary information for additional details) and compared with citrate-modified AuNPs (citrate-AuNPs) with negative charges (BBI Solutions OEM Ltd., Cardiff, UK) in terms of their effects on Aβ growth. Both types of nanoparticles were 40 nm in size, with the same molar concentration. To maintain the same total surface area, 75 pM of 40-nm AuNPs were used as equivalent to the total area of 50 pM of 50-nm AuNPs. First, we captured dark-field and TEM images after 6 hr and 48 hr of incubation, to detect clustering of AuNPs and determine the structure of Aβ co-aggregates with AuNPs (Fig. 4). In the dark-field images, after incubation for 6 hr, no clear differences between the two samples were observed, but the color and size of the aggregates could be discriminated after 48 hr of incubation. Citrate-AuNPs formed larger Aβ aggregates by gathering more peptides and AuNPs together, whereas amine-AuNPs formed smaller aggregates. The TEM data (Fig. 4a) showed that Aβ and clustered amine-AuNPs formed small amorphous aggregates; it appears that the Aβ peptides could not form protofibrils. As mentioned, there were six negatively charged residues (D1, E3, D7, E11, E22, and D23) and three positively charged residues (R5, K16, and K28) in Aβ sequence, and therefore the electrostatic interactions between positively charged AuNPs and Aβ would be stronger, which could result in electrolyte-induced aggregation followed by misfolding of peptides, inhibiting further fibrillization^14^,^33^. Tight interactions between AuNPs and Aβ could limit the structural flexibility of Aβs which is necessary for conformational conversion, and inhibit accommodation of other Aβ monomers on the surface or in solution^12^,^34^. In other words, Aβ peptides are strongly adsorbed onto the surface of amine-AuNPs and conformational conversion of these surface-bound Aβs would be hindered, resulting in retardation of Aβ aggregation. As incubation time increased, the TEM image showed formation of fibrils with densely packed amine-AuNPs, shown by orange coloring in the correlated dark-field image (Fig. 4b). However, after 6 hr of incubation of Aβ and citrate-AuNPs, protofibrils or short fibrils were produced around citrate-AuNPs without AuNP clustering (Fig. 4a). The surface of citrate-AuNPs was mostly covered by surface-bound 40-nm Aβ peptides, and these citrate-AuNPs could then act as nucleation seeds of further aggregation by increasing the local concentration of Aβs, with fewer constraints on conformational conversion. Based on the short lag phase, Aβ aggregation was accelerated by agglomeration of AuNPs. Similar results were observed for the citrate-AuNPs in the dark-field and TEM images as were observed for the previously mentioned co-incubation with 50-nm AuNPs, forming Aβ and AuNPs co-aggregates in close proximity (Fig. 4). The sizes of amine-AuNP and citrate-AuNP aggregates increased from ~55 and ~46 to ~85 and ~62 nm, respectively, after 30 min of incubation (Supplementary Fig. S3b). The net charge of Aβ is negative at a physiological pH (pI of Aβ = pH 5.2)^35^, so the surface charge of nanoparticles will be negative when Aβ monomers were attached. Given these results, amine-AuNPs interacted with Aβ peptides through electrostatic interactions between the negatively charged amino acids and the functional groups on the surface of AuNPs. In addition, amine-AuNPs covered by Aβ peptides would have negative charges, leading to clustering of amine-AuNPs owing to the strong electrostatic interactions. In contrast, citrate-AuNPs have negatively charged surfaces, and a reason for Aβ binding to citrate-AuNPs is Aβ-AuNP complex formation involving the replacement of the citrate groups on AuNPs with Aβ peptides and the direct attachment of AuNPs to Aβ peptides^9^.

### Detection of interaction sites in Aβ and secondary structures of Aβ-AuNP co-aggregates

**Figure 5:**
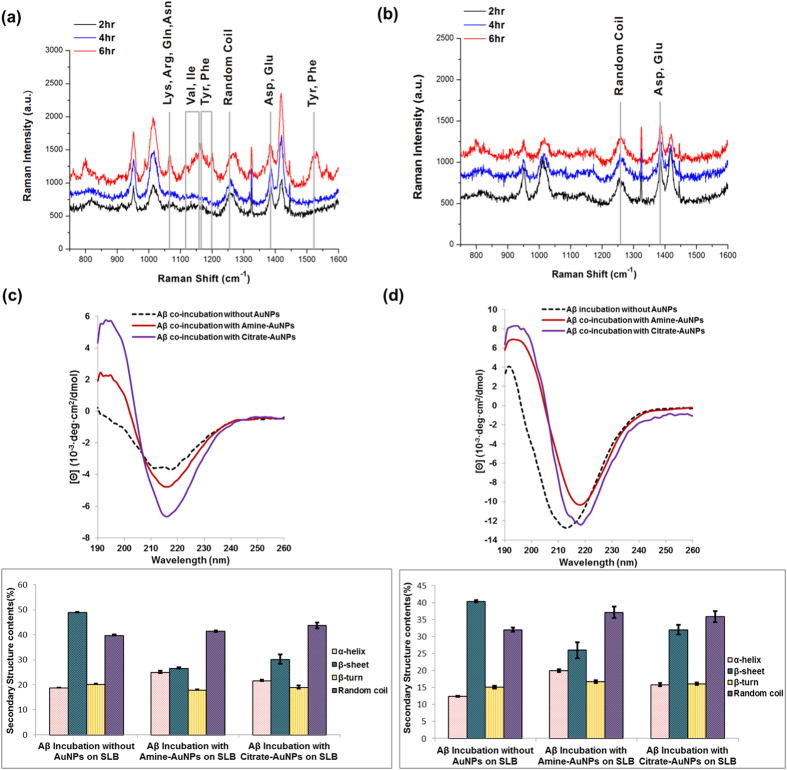
Study on the interactions between Aβ and differently-charged AuNPs and secondary structural analysis of Aβ aggregates. (**a**) The SERS spectra from time-lapse incubation of Aβ and amine-AuNPs. (**b**) The SERS spectra of Aβ on the surface of citrate-AuNPs with varying incubation time. The CD spectra show the secondary structures of Aβ aggregates incubated with AuNPs for (**c**) 6 hr and (**d**) 48 hr. The error bars were calculated with three independent samples.

To reveal the structural changes in Aβs on the surface of AuNPs, time-lapse SERS signals were collected during incubation. As seen in Fig. 5a, although the SERS signals of amine-AuNPs did not differ after incubation for 2 hr and 4 hr, predominantly showing CH_2_ symmetric rocking, CH_2_, CH_3_ deformation, the S = O of Met, and the COO^–^ stretching of Asp and Glu, the results indicate that some residues were in close contact with the surface of AuNPs. Amino acids containing aromatic residues such as Phe and Tyr, and nonpolar residues such as Met, Val, and Ile could directly interact with the surface of the metal. The hydrophobic residues (Phe, Ile, Val, and Gln) and Lys of Aβ peptides were likely bound to the surface of amine-AuNPs, which could hinder conformational changes into cross β-sheet structures. As incubation time increased to 6 hr, amine-AuNPs aggregated due to the negative charge of Aβ residues, followed by increased interaction of Aβ peptides on the surface of clustered AuNPs with stronger SERS signals. In contrast, as shown in Fig. 5b, the SERS peaks of Aβ peptides on the surface of citrate-AuNPs were independent of the incubation time; they showed some peaks indicating CH_2_ symmetric rocking, CH_2_, CH_3_ deformation, the S = O of Met, and the COO¯ stretching of Asp and Glu due to formation of Aβ and citrate-AuNP complexes through exchange of citrate ligands for negatively charged Aβ residues. Both amine-AuNPs and citrate-AuNPs clearly showed peaks at 1254 cm^–1^ after 2 hr incubation, representing the formation of random coil Aβ peptide structures. It could be concluded that the Gln and Lys residues and hydrophobic residues such as Phe, Ile, and Val of Aβ peptides were preferentially bound to the surface of amine-AuNPs, which may have inhibited conformational change of Aβ into cross β-sheet structures, resulting in spherical aggregates after 6 hr of incubation. Compared to the amine-AuNP case, Aβ peptides on the surface of citrate-AuNPs showed less diverse SERS peaks with random coil feature as well as Asp and Glu residue features. The results indicate the easier conformational change of the Aβ peptides to β-sheet structures within the same incubation time is possible for the citrate-AuNP case.

The CD results (Fig. 5c) shows that amine-AuNPs co-incubation produced fewer β-sheet structures than co-incubation with citrate-AuNPs for 6 hr or 48 hr. As shown in the TEM images in Fig. 4, after 6 hr, amine-AuNPs induced small amorphous Aβ aggregates, with fewer α-helix or β-sheet structures and more random coils, and, after a longer incubation, fibril structures were branched from the clustered amine-AuNPs. Those amorphous aggregates consisted of β-sheet structures and might represent intermediate stages of Aβ elongation and aggregation. This result was concordant with the CD spectra, which showed that the number of β-sheet structures increased as incubation time increased. Citrate-AuNPs acted as nucleation seeds, reducing the lag time, so protofibrils were formed after 6 hr and further aggregation occurred as incubation time increased, producing more β-sheet structures. Finally, the surface charge of nanoparticles can greatly influence Aβ growth and control their conformation, causing changes in secondary structures.

### Observing Aβ aggregates formed with different shapes of Au nanostructures

**Figure 6:**
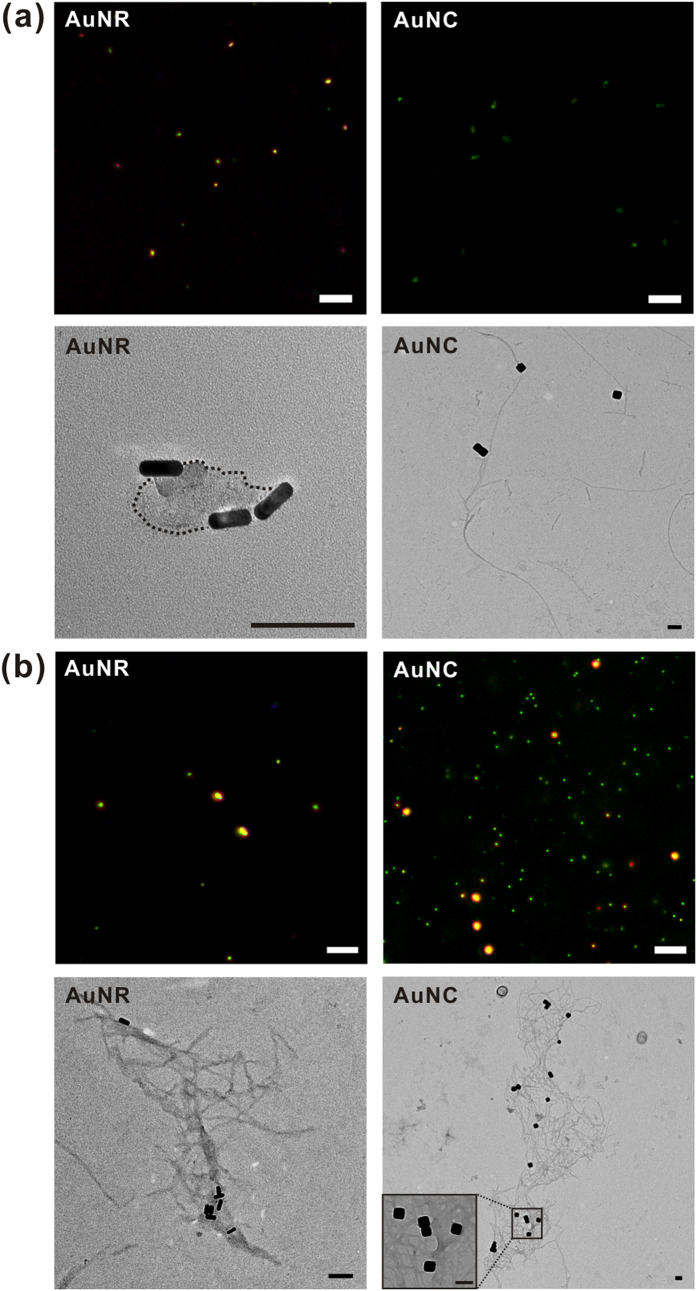
The dark-field and TEM images for Aβ aggregates incubated with AuNRs and AuNCs on the brain SLB. The image were obtained after (a) 6-hr incubation and (**b**) 48-hr incubation. The scale bars of the dark-field images are 10 μm and those of the TEM images are 100 nm.

Next, how particle shape affects Aβ aggregation on the brain SLB was studied by comparing spherical AuNPs, anisotropic gold nanorods (AuNRs) and multi-facted gold nanocubes (AuNCs). All these particles were modified with amine functional groups, providing positively charged surfaces. It should noticed that tuning the aspect ratio of AuNRs or truncating AuNCs produces resonance peaks in the near-IR region (700–1300 nm), useful range for *in vitro* sensor and *in vivo* imaging/therapeutic applications^36^,^37^. We synthesized AuNRs and AuNCs with one side of a similar length, and the long axis of the AuNRs and the edge of the AuNC were approximately 50 nm, to facilitate comparison of their structural effects. The morphology and color of Aβ aggregates with AuNRs or AuNCs were examined via dark-field microscopy and TEM (Fig. 6). AuNRs generate LSPR effects at two distinct wavelengths that correspond to the longitudinal mode and the transverse mode in the near-IR region (700–1300 nm) at an appropriate aspect ratio^38^,^39^. In this study, the aspect ratio of AuNRs was approximately 3.17, showing a longitudinal mode LSPR peak in the near-IR region^39^, so light was scattered at approximately 520 nm owing to the transverse mode employed for dark-field imaging (Supplementary Fig. S5). The AuNRs had a short axis of 13.55 nm and a long axis of 42.96 nm; the length of the edge of the AuNCs was 51.05 nm. The particles were uniform, and green colors were obtained under dark-field microscopy (Supplementary Fig. S5). In this experiment, the total surface area of the AuNRs and the AuNCs remained the same via adjustment of the concentration of the nanoparticles. When AuNCs are compared to AuNRs, larger aggregates were formed on AuNCs than on AuNRs mainly because AuNCs have a larger effective surface area with more isotropic structures than AuNRs. However, it should be also noted that, although spherical AuNPs are more isotropic, the aggregates grown on AuNPs displayed poorer structure and lower percentage of β-sheet than AuNCs. This is because the β-sheet-aggregation-inducing amino acids in Aβ peptide, Phe, Tyr, Met, Val and Ile, closely interacted with AuNPs (Fig. 5a), and this hindered the formation of β-sheet-stacking-based Aβ aggregates while AuNCs did not interact with β-sheet-aggregation-inducing amino acids.

The dark-field scattering color of Aβ peptides, co-incubated with AuNRs for 6 hr, was green, but the color was changed to orange after 48 hr incubation (Fig. 6a). This indicates that AuNRs were aggregated after 6 hr of involvement in Aβ growth. To detect aggregate states, we obtained the TEM images for the same samples, and concluded that AuNRs had relatively weak interactions with Aβ peptides, resulting in smaller aggregates after 6 hr of incubation and a few fibrils after 48 hr of incubation (Fig. 6). It was reported that cetyltrimethylammonium bromide (CTAB) specifically binds to {100} faces, along the length of rods and forms positively charged surface of AuNRs^40^, and it is likely that Aβ was preferentially bound to the long axis surface of AuNRs. When AuNRs of a different aspect ratio were incubated with Aβ for 48 hr, there were no noticeable changes in the morphology of the Aβ co-aggregates (Supplementary Fig. S6).

Figure 6a,b show the results of incubation of AuNCs with Aβ for 6 hr and 48 hr, respectively; the distinct scattering signals in the dark-field images resulted from the strong LSPR properties of AuNCs^41^. Although the edge length of the AuNCs is similar to the long axis length of the AuNRs, the AuNCs have a larger effective surface area with more isotropic structures than AuNRs. Long Aβ fibrils were observed within 6 hr with AuNCs. In the dark-field images, the color changed to yellowish green or yellow after 6 hr of incubation, followed by a more red-shifted to orange color in some cases after 48 hr incubation. In addition, the morphology of Aβ-AuNC co-aggregates was observed to be networks with distinguishable and entangled fibrils that would likely be rich in β-sheet structures. Aβ could bind to AuNCs in different directions, facilitating Aβ growth on the surface of AuNCs. Thereafter, Aβ peptides were grown on six-faceted AuNCs, resulting in a more rapid nucleation process; Aβ fibrils were interwoven, resulting in networks of fibrils with AuNCs (Fig. 6b).

### SERS and CD measurement for detecting interaction sites and secondary structures of Aβ aggregates

**Figure 7:**
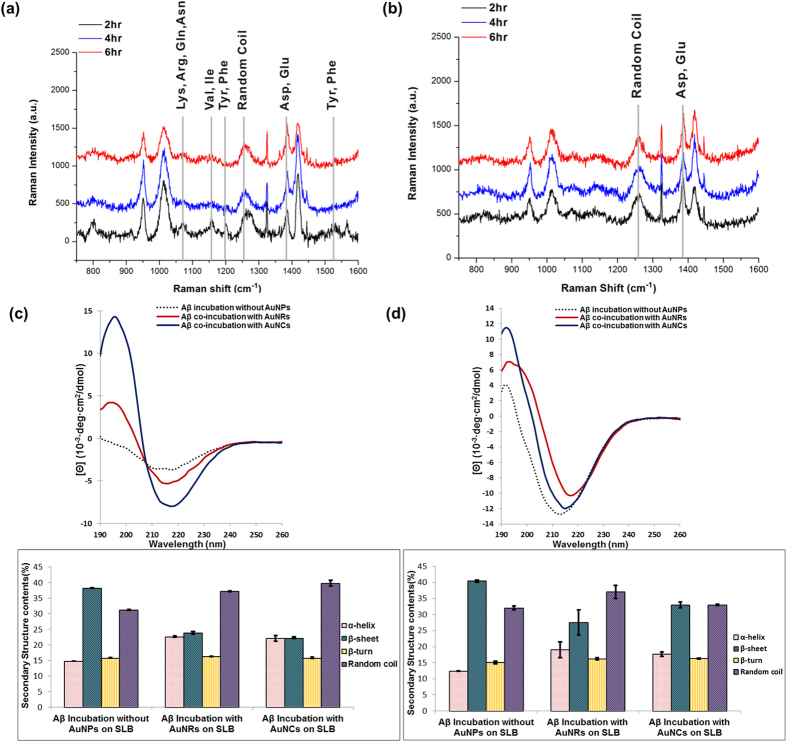
Analyses on the interactions between Aβ and differently-shaped nanoparticles and secondary structural analysis of Aβ aggregates. The SERS signals were measured after time-lapse incubation for (**a**) AuNRs and (**b**) AuNCs. The CD spectra show the secondary structures of Aβ aggregates incubated with AuNRs or AuNCs for (**c**) 6 hr and (**d**) 48 hr. The error bars were calculated with three individual replicates.

We then investigated both their secondary structural features of Aβ aggregates and interaction between Aβ and the surface of Au nanostructures in more details (Fig. 7). The structure of Aβ aggregates is dependent on the initial local concentration. Hence, the adsorption isotherms were obtained for AuNRs and AuNCs with spherical AuNPs functionalized with amine groups (Supplementary Fig. S3d). Incubation with AuNCs differed from that with AuNRs or AuNPs in that the curve showed a maximum equilibrium surface concentration, and the adsorbing and desorbing constant for Aβ peptides on AuNCs reached equilibrium in the same incubation period. The shape of the adsorption isotherm indicated an adsorption affinity for peptides, and the maximum amount of adsorbed molecules and binding affinity could be determined after equilibrium was reached^42^. This result also supported that the initial concentration of Aβ peptides on the surface could be an important factor^15^. Both AuNRs and AuNCs possess positively charged surfaces but AuNRs and AuNCs interacted differently with Aβ at the beginning of incubation, as seen in Fig. 7a,b. In the case of AuNRs, several peaks with low intensity, stemming from positively charged or polar residues such as Lys, Arg, Gln, and Asn, and aromatic or nonpolar residues such as Phe, Tyr, Val, and Ile, were detected after 2 hr of incubation, whereas those peaks disappeared as incubation time increased. The SERS signals showed that CH_2_ symmetric rocking, CH_2_, CH_3_ deformation, S = O of Met, and the COO^–^ stretching of Asp and Glu remained throughout the incubation process, which implies that Aβ peptides were closely bound to the surface of AuNRs within a short time, followed by formation of random coil structures. The residues including Phe, Ile, Val, Gln, and Lys of Aβ peptides were likely bound to the surface AuNRs, and this may inhibit conformational changes into cross β-sheet structures, resulting in rather spherical aggregates after 6 hr incubation. On the other hand, the representative peaks near 1254 cm^–1^ were detected for random coil structures after incubation of Aβ peptides with AuNCs, and no significant incubation-time-dependent changes were observed. The Aβ peptides on the surface of AuNCs only showed several peaks of random coil structures and Asp and Glu residues, and the Aβ peptides were changed to form β-sheet structures after 6 hr incubation. In addition, we could discern secondary structural changes in CD measurements after 6 hr and 48 hr of incubations (Fig. 7c,d), strictly correlated with the morphology observed in TEM images. When AuNRs and AuNCs were co-incubated with Aβ for 6 hr, AuNCs accelerated Aβ fibrillization, producing fibrils bound to AuNCs-more β-sheet structures were observed for the AuNC case than the AuNR co-incubation, which showed fewer β-sheet and more α-helix structures (Fig. 7d). As incubation time increased, Aβ formed fibrils on both samples, but the quantity of fibrils and their structural characteristics were different (Fig. 7d). The networks of Aβ fibrils with AuNCs were mostly composed of β-sheet and random coil structures, whereas the few fibrils that were bundled with AuNRs contained more random coil structures than β-sheet structures, although the amount of β-sheet structures was increased.

### Cell viability assay with SH-SY5Y neuroblastoma cells

**Figure 8:**
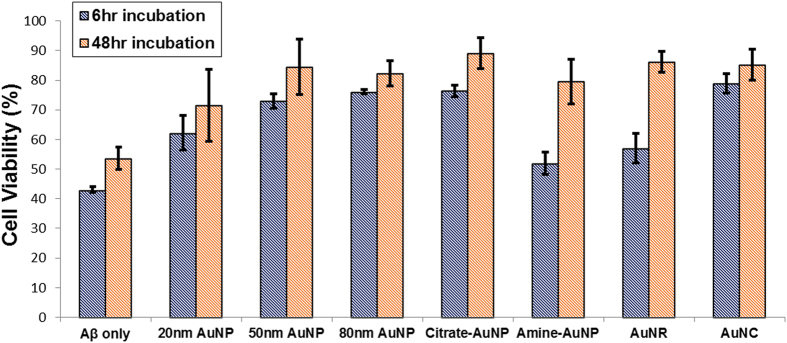
Cell viability assay of Aβ aggregates formed after 6-hr and 48-hr incubation with SH-SY5Y neuroblastoma cells using CCK-8 assay. After 6-hr and 48-hr incubation of Aβ and Au nanostructures on the brain SLB, the collected Aβ aggregates were incubated with SH-SY5Y cells at a concentration of 1.0 × 10^4^ cells/well in 96-well plates. The error bars were calculated with three individual replicates.

We next studied the cytotoxicity of NP-Aβ aggregates on neuroblastoma cells, and SH-SY5Y neuroblastoma cells were used to perform a cell viability assay using CCK-8 assay kit (Fig. 8). It was shown that Aβ oligomers are more toxic than Aβ fibrils or plaques, inducing acute cell death^2^,^3^. Self-assembled Aβ oligomers cause ion dyshomeostasis, membrane permeabilization, oxidative stress to the cell membrane, and synaptotoxicity, and larger Aβ oligomers or spherical Aβ assemblies of approximately 15 nm could be the elusive toxic species^43^. After Aβ peptides were co-incubated with Au nanostructures for 6 hr and 48 hr, the differently structured aggregates were formed. As shown in Fig. 8, after 6 hr of incubation, the Aβ structures without AuNPs were highly toxic, with 43% cell viability, and the Aβ aggregates with amine-AuNPs and AuNRs yielded approximately 51% and 57% cell viabilities, respectively. Spherical Aβ aggregates incubated with amine-AuNPs or AuNRs showed more toxicity to SH-SY5Y cells than the fibrils formed with other types of Au nanostructures. When fibrils with a wide range of lengths were formed with AuNCs and citrate-modified AuNPs, cell viability was increased. As NP-Aβ co-incubation time increased, the toxicity of Aβ aggregates decreased. However, after 48 hr of incubation without Au nanostructures or with 20-nm AuNPs, cell viability was less than 70%. In the case of 20-nm AuNP co-incubation of Aβ on the brain SLB for 48 hr, protofibrils and short fibrils that are toxic to neuroblastoma cells were dominantly formed. In contrast, 50-nm AuNPs, 80-nm AuNPs and citrate-AuNPs induced plaque-like Aβ aggregates. AuNCs also produced the networks of fibrils. Amine-AuNPs and AuNRs induced the formation of fibril bundles while longer fibrils were formed with amine-AuNPs. Au nanostructure-induced mature fibrils or plaque-like structures of Aβ aggregates resulted in the low toxicity (>80% cell viability).

## Discussion

We showed how the size, shape and surface charge of nanoparticles influence Aβ aggregation and fibrillization on the brain SLB and studied the cytotoxicity of AuNP-Aβ co-aggregates on neuroblastoma cells. Aβ peptides interacted with anionic lipids in the lipid bilayer, showing a macromolecular crowding effect and folding into structures rich in β-sheets on the SLB. It should be noticed that the size, shape and surface charge of nanoparticles are tunable, and these nanoparticles could be used as drug carriers, photothermal and photodynamic therapeutic tools or inhibitors of Aβ aggregates. Further, Au nanostructures have great utility as imaging tools, in that they generate LSPR effects at specific wavelengths, allowing us to obtain a variety of optical data on the interactions between peptides and nanoparticles. Our results offer a systematic and fundamental understanding on Aβ aggregation with nanoparticles on a fluid membrane platform and facilitate further development of tools for diagnosis and cure of Alzheimer’s disease using nanostructures.

## Methods

Procedures for initial preparation of Aβ peptides and preparation of lipid vesicles and the supported lipid bilayer (SLB) were described in a previous paper^9^.

### Co-incubation of Aβ with AuNPs and dark-field imaging of Aβ aggregates

The dark-field chambers containing SLB were washed with 600 μL of 10 mM PB (pH 7.4) by flowing through the space between two glasses to optimize conditions for Aβ fibril growth. AuNPs (10 μL) was mixed with 100 μL of Aβ solution just before use. The AuNP concentrations were varied in accordance with the surface area of each particle. Subsequently, 110 μL of the resulting solution was injected into the chamber, and the samples were incubated at 37 °C and 5.0% CO_2_ for 6 hr and 48 hr, respectively. Dark-field microscopy was performed using a 200 inverted microscope (Carl Zeiss, Oberkochen, Germany) equipped with a dark-field condenser (NA = 1.4, oil immersion) and white light illumination from a 100-W halogen lamp. Images were captures using a 40× objective lens (NA = 0.8) (Axiovert 200M, Carl Zeiss, Oberkochen, Germany).

### TEM imaging of Aβ aggregates via negative staining

To prepare TEM specimens, air was injected though the chamber inlet and the solution was then pushed out through the chamber outlet. 10 μL of this solution was dropped onto a TEM grid and after 10 min, the remaining solution was soaked up from the edge of the grid using filter paper. This sample was dried at room temperature overnight before imaging. The specimen was then stained with 10 μL of 2% uranyl acetate solution in deionized water for 1 min, and the staining solution was drawn away from the edge of the grid with a filter paper. The TEM grid was washed with 10 μL of deionized water 3 times and dried overnight at room temperature. Then, we observed the sample using the transmission electron microscope (JEOL-JEM 2100, JEOL, Tokyo, Japan) in the National Center for Inter-University Research Facilities (NCIRF).

### Circular dichroism measurement

We used a circular dichroism (CD) spectrometer (Chirascan Plus, Applied Photophysics, UK) to detect secondary structural changes in Aβ aggregates. To obtain the signal of Aβ aggregates *in situ* without disruption of the brain SLB and Aβ aggregate structures, SLB was fabricated on quartz cells. First, quartz cells were immersed in piranha-etching solution (3:1 = concentrated sulfuric acid/30% hydrogen peroxide) for 40 min, thoroughly rinsed with deionized water, and then dried with a stream of nitrogen. Small unilamellar vesicles (SUV, 100-nm diameter) of 100 mol% brain total lipid extract were mixed with 150 mM PBS (1:1 (v/v)), and 400 μL of the resulting solution was added into a piranha-etched quartz cell. After 30 min of incubation at room temperature, the quartz cell was washed with 10 mM PB (pH 7.4) to remove excess vesicles and to provide appropriate conditions for Aβ growth. Finally, 400 μL of the Aβ solutions including AuNPs were carefully injected into the quartz cells. The quartz cells were immediately sealed and incubated at 37 °C and 5.0% CO_2_ for 6 hr and 48 hr. The secondary structure was analyzed using the program CDNN (Applied Photophysics, Leatherhead, Surrey, UK).

### Surface-enhanced Raman scattering (SERS) measurement of Aβ-attached AuNPs

Silicon sticker chambers (2.5 mm in diameter) were fixed to 25 mm × 25 mm microscopic cover glasses (Fisher Scientific, Pittsburgh, PA, USA). 10 μl of SUV solution (1 mg/ml lipid concentration in PBS) was injected into each chamber and the chambers were incubated for 40 min. Then, the coverglasses were immersed in a deionized water bath and excess SUV suspension was removed with flowing water. Then, the glasses were placed on a Petri dish, and the water level was adjusted to match the height of the sticker chamber. Co-incubated samples of Aβ peptides and AuNPs were prepared immediately before SERS measurements were taken; the concentration was identical to that used for the dark-field and TEM measurements. Lastly, 3 μl of solution was removed from each chamber and 3 μl of the mixed solution of Aβ and AuNPs was injected. SERS signals were obtained after 2 hr, 4 hr, and 6 hr of incubation using a Renishaw inVia microscope equipped with a Leica microscope and the Renishaw WiRE 3.1 software. A 633-nm laser (HeNe laser, 10 mW) was used to produce Raman scattering under a 50× objective lens (N/A = 0.75) with a 10-sec data acquisition period. Peak assignment was demonstrated in Supplementary Table S1.

### Cell viability assay

SH-SY5Y cells were purchased from the Korean Cell Line Bank (KCLB, Seoul, South Korea) and cultured in 10% fetal bovine serum (FBS)-supplemented Minimum Essential Media (MEM) (Gibco, USA) with 100 U/ml penicillin–100 μg/ml streptomycin (Gibco, USA) at 37 °C and 5% CO_2_. SH-SY5Y cells were plated at a concentration of 1.0 × 10^4^ cells/well in 96-well plates with 100 μL of media and incubated overnight. Aβ monomers (25 μM) were incubated on SLB with seven different types of gold nanostructures with identical total surface areas, as descried above. A sample containing 25 μM of Aβ monomers without Au nanostructures was also incubated for 6 hr and 48 hr as a control. Then, each specimen was collected by peeling it off the SLB and centrifugation of the resulting solution for 1 min. 10 μL of collected Aβ aggregates was placed onto each well of the 96-well plate, and the samples were incubated for 8 hr at 37 °C and 5% CO_2_. To test the cytotoxicity of Aβ aggregates to neuroblastoma cells, we used a CCK-8 assay kit (Dojindo Molecular Technologies, Inc., Rockville, MD, USA). After incubation of Aβ aggregates with SH-SY5Y cells, 10 μL of CCK-8 solution was added to each well and absorbance at 450 nm was measured after 1 hr of incubation using a Synergy™ MX (BioTek Instruments, Inc., Winooski, VT, USA).

## Additional Information

**How to cite this article**: Kim, Y. *et al*. How Do the Size, Charge and Shape of Nanoparticles Affect Amyloid ß Aggregation on Brain Lipid Bilayer? *Sci. Rep*. **6**, 19548; doi: 10.1038/srep19548 (2016).

## Supplementary Material

Supplementary Information
