# Erratum: How Do the Size, Charge and Shape of Nanoparticles Affect Amyloid β Aggregation on Brain Lipid Bilayer?

**DOI:** 10.1038/srep22173

**Published:** 2016-03-04

**Authors:** Yuna Kim, Ji-Hyun Park, Hyojin Lee, Jwa-Min Nam

Scientific Reports 6: Article number: 19548; 10.1038/srep19548 published online: 01192016; updated: 03042016.

This Article contains a typographical error in the Author Contributions Statement.

“J.-M.N. conceived the initial idea. J.-M.N. and Y.K. designed experiments. Experiments were performed by Y.K. and J.-H.P. under the guidance of J.-M.N. and Y.K. and J.-M. analyzed the data. N.H.L. provided technical assistance. J.-M.N. and Y.K. wrote the paper.”

should read:

“J.-M.N. conceived the initial idea. J.-M.N. and Y.K. designed experiments. Experiments were performed by Y.K. and J.-H.P. under the guidance of J.-M.N. Y.K. and J.-M.N analyzed the data. H.L. provided technical assistance. J.-M.N. and Y.K. wrote the paper.”

In addition, the Author Contributions Statement is duplicated in the Acknowledgements.

The Acknowledgements should read:

“This work was supported by a National Research Foundation (NRF) of Korea (Grant No. 2011-0018198) and BioNano Health-Guard Research Center funded by the Ministry of Science, ICT & Future Planning (MSIP) of Korea as Global Frontier Project (H-GUARD_2013M3A6B2078947).”

