# Supplementary material for: How Do the Size, Charge and Shape of Nanoparticles Affect Amyloid β Aggregation on Brain Lipid Bilayer?
Source: Sci Rep. 2016 Jan 19;6:19548. doi: 10.1038/srep19548 (PMC4726094; doi:10.1038/srep19548)
Supplement: Supplementary Information [file srep19548-s1.pdf]

Supplementary Information

How Do the Size, Charge, and Shape of Nanoparticles Affect Amyloid β Aggregation on Brain Lipid Bilayer?

Yuna Kim, Ji-Hyun Park, Hyojin Lee, and Jwa-Min Nam*

Department of Chemistry, Seoul National University, 599 Gwanak-ro, Gwanak-gu, Seoul 151-742, South Korea

* Correspondence should be addressed to J.-M. N. (jmnam@snu.ac.kr)

Tel: (+82) 2-880-6816, Fax: (+82) 2-888-6815

This file includes:

Supplementary Figures 1-6

Supplementary Table 1

Supplementary Methods

Supplementary Figures

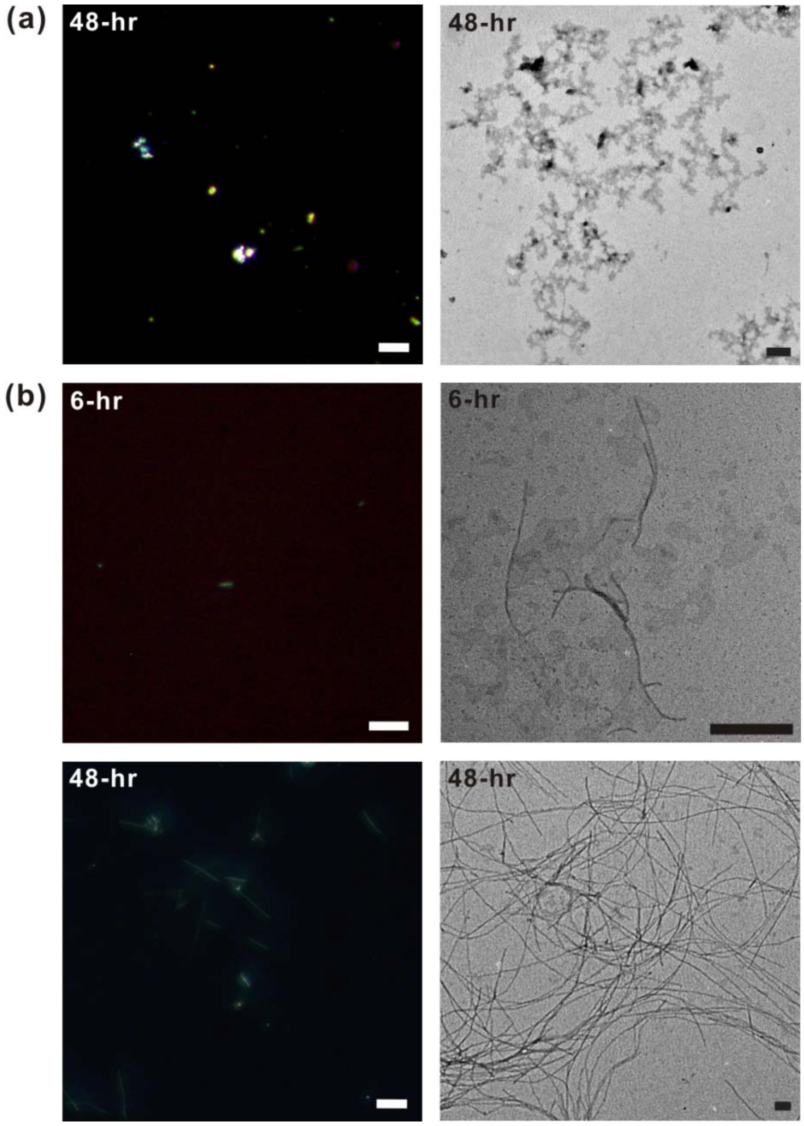

Supplementary Figure S1. The dark-field and TEM images after incubation of Aβ (a) without SLB and (b) with SLB. (a) Without SLB, Aβ could form radomly structures aggregates rather than β-sheet rich fibrils. Light scattering in dark-field images was also irregular due to the variation of the size and the shape of aggregates. (b) After 6-hr incubation, protofibrils, and short fibrils were formed and they became muture fibrils after 48-hr incubation. Scale bars are 10 μm for dark-field images and 200 nm for TEM images.

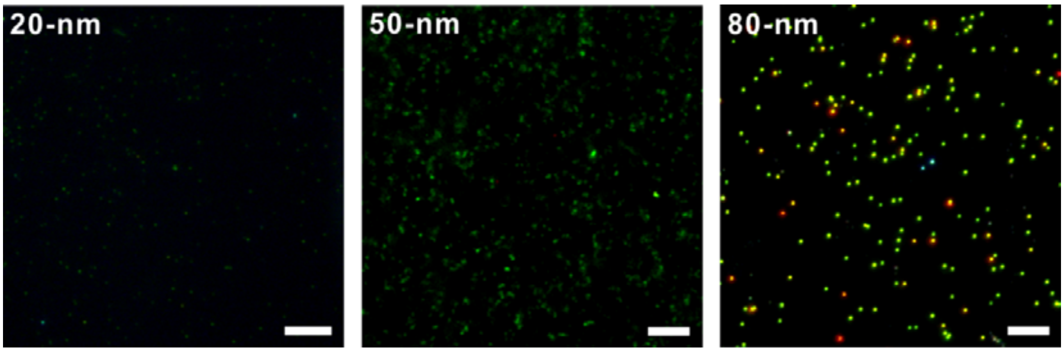

Supplementary Figure S2. The dark-field images of 20-nm, 50-nm and 80-nm AuNPs. 20-nm and 50-nm AuNPs showed only green colors in the dark-field images, whereas 80-nm AuNPs also scattered yellow and red colors in several spots. The overall intensity from each particle becomes stronger as particle size increases. Scale bars are 10 μm for all the images.

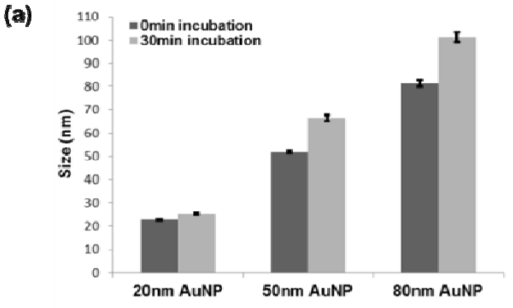

Size(nm)	0min incubation	30min incubation
20nm AuNP	22.79±0.22	25.34±0.33
50nm AuNP	51.87±0.31	66.50±1.21
80nm AuNP	81.41±1.49	101.20±2.07

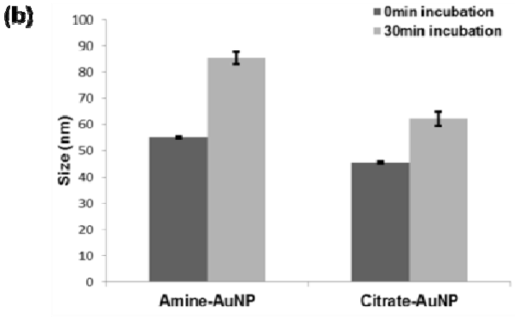

Size(nm)	0min incubation	30min incubation
Amine-AuNP	55.16±0.42	85.39±2.47
Citrate-AuNP	45.66±0.31	62.28±2.78

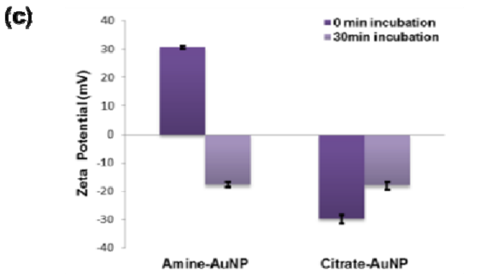

Zeta Potential (mV)	Amine-AuNP	Citrate-AuNP
0 min incubation	30.73	-29.8
30min incubation	-17.67	-18.03

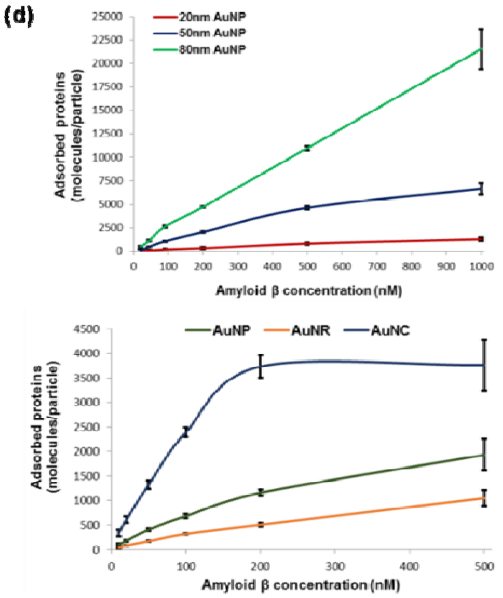

Supplementary Figure S3. Difference in particle **size and charge after 30-min incubation of Aβ and analysis on the interactions between Aβ and AuNPs.** The sizes of Aβ and AuNP aggregates after 30-min incubation with (a) different sizes of AuNPs and (b) differently surface-charged AuNPs. (c) The zeta potential data after 0-min and 30-min incubation of Aβ with amine-AuNPs and citrate-AuNPs, respectively. (d) The adsorption isotherms of Aβ and AuNPs after 30-min co-incubation with different types of AuNPs.

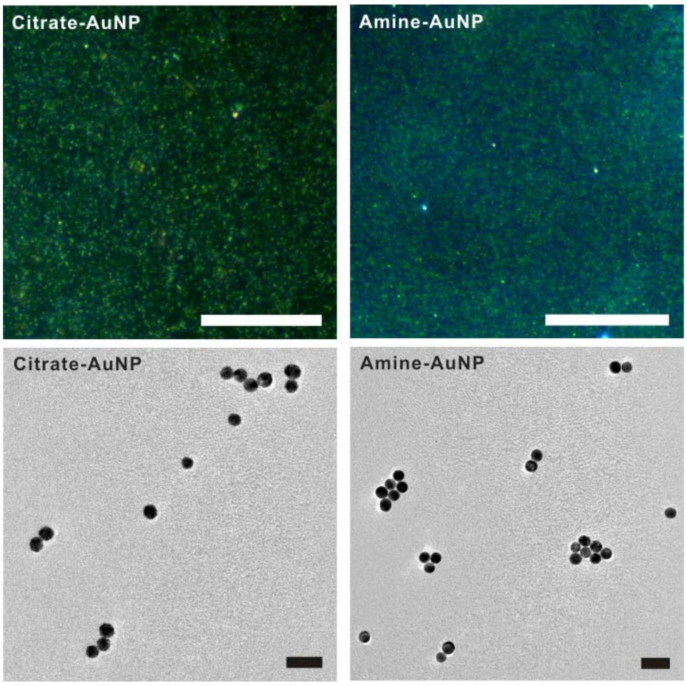

Supplementary Figure S4. The dark-field and TEM micrographs of citate-AuNPs (left) and amine-AuNPs (right). The particle diameter for amine-AuNPs and citrate-AuNPs were 39.06±2.06 nm and 37.67±1.52 nm, respectively. The sizes were measured from the TEM images. Scale bars in the dark-field images are 100 μm and those in TEM images are 100 nm.

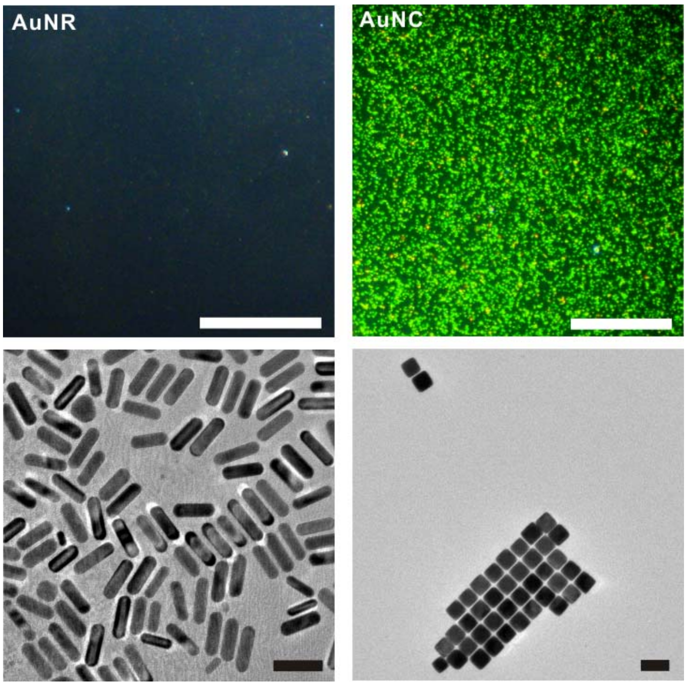

Supplementary Figure S5. The dark-field and TEM images of AuNRs (left) and AuNCs (right) to verify the particle size and distribution. The scale bars in dark-field data are 100 μm, and the TEM images have 100 nm scale bars.

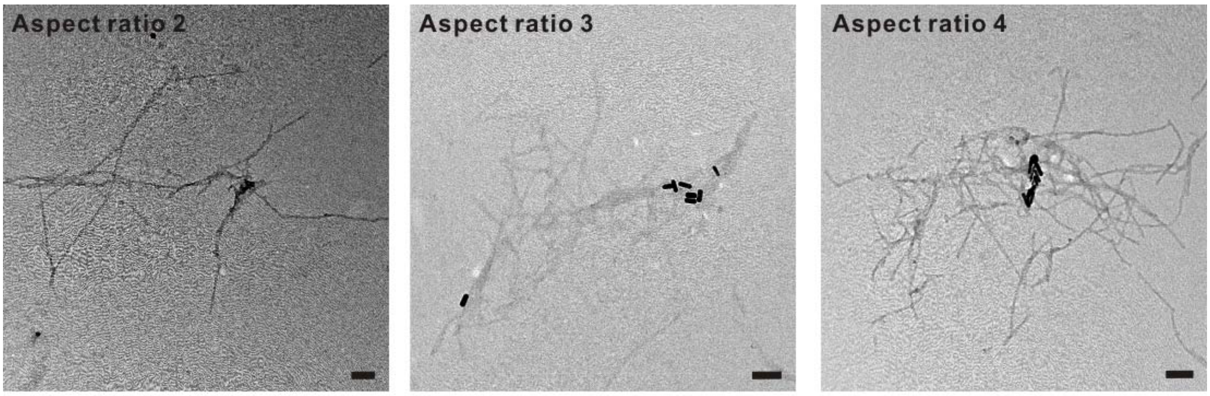

Diameter(nm)	Short axis (nm)	Long axis (nm)	Aspect ratio
AuNR (1:2)	18.84±1.66	37.53±4.79	1.98±0.14
AuNR (1:3)	13.55±0.61	42.96±3.23	3.17±0.28
AuNR (1:4)	13.17±1.21	52.47±2.82	3.98±0.44

Supplementary Figure S6. TEM images obtained after 48-hr co-incubation of Aβ with three types of AuNRs. The scale bars are 100 nm.

Table 1. Assignment of bands in SERS spectra of Amyloid β

Wavenumber (cm^{-1})	Assignment
763	Ala
827	Tyr
952	CH_2 symmetric rock
1002	Phe
1013	S=O, methionine sulfoxide
1066	[C-C and C-N stretch], Lys, Arg, Gln, Asn
1116	Val, Ile + [C-Cα and C-N stretch]
1153	[C-N] + Ile
1160	Val, Ile
1167	Tyr, Phe
1201	Tyr, Phe
1235	amide III in β-sheet
1254	amide III random
1298	amide III in α-helix
1318	amide III and CH_2 twist/wag
1343	[Cα-H bend + Cα-C stretch] + CH_2 twist/wag
1386	COO^- symmetric stretch in Asp, Glu
1420	CH_2 scissor, CH_3 deformation
1449	CH_2 scissor, CH_3 deformation
1575	Phe
1604	[Zn-histidine], Phe, Tyr-
1624	C=O bound to metal

Supplementary Table 1. The Raman bands in the SERS spectra of Aβ. The band assignments are based on references 35-37.

Supplementary Methods.

Synthesis of Spherical Gold Nanoparticles (AuNPs). We purchased citrate modified AuNPs whose sizes were 20-nm, 40-nm, 50-nm, and 80-nm AuNPs, and amine modified AuNPs was synthesized as following. 5 mL of 200 mM hexadecyltrimethylammonium chloride (CTAC) and 5 mL of 0.5 mM gold chloride trihydrate ($HAuCl_4$) were added to a 50mL round-bottom-flask (r.b.f) at 30℃ under magnetic stirring at 500 rpm. 0.45 mL of 20 mM sodium borohydride ($NaBH_4$) was directly added to this solution and mixed for 2 min resulting in color change of seed solution from orange to brownish yellow and the solution was left undisturbed at 30 ℃ for 1 hr. Growth solution was prepared in two 50 mL r.b.f. labeled A and B. 5 mL of 200 mM CTAC, 4.625 mL of deionized water, 250 μL of 10 mM $HAuCl_4$, 10 μL of 10 mM NaBr, and 90 μL of 40 mM L-ascorbic acid were added to both 50mL r.b.f.s repectively. Finally, 30 μL of seed solution was added to the growth solution in A under magnetic stirring at 80 rpm until the solution color turned red (~30 s). Then, 100 μL of the solution in A was transferred to growth solution B under magnetic stirring at 80 rpm for 30 s and kept undisturbed at 26 ℃ for 12 hr. The resulting red color solution is indicative of AuNPs having a diameter of 40 nm. Then, the solutions were centrifuged twice at 5000 rpm for 10 min.

Synthesis of Gold Nanorods (AuNRs). 5mL of 200mM hexadecyltrimethylammonium bromide (CTAB) and 5 mL of 0.5 mM $HAuCl_4$ were added to a 50 mL r.b.f in a water bath at 25 ℃ under magnetic stirring at 500 rpm. Then, 0.6 mL of 10 mM ice-cold $NaBH_4$ was added and mixed for 2 min. The resulting brownish-yellow seed solution left undisturbed in a 25 ℃ water bath for 2 hr thereafter 5 mL of 200 mM CTAC, 5 mL of 1 mM $HAuCl_4$, 0.2 mL of 4 mM silver nitrate($AgNO_3$), and 70 uL of 78.8 mM L-ascorbic acid were added in sequence with gentle shaking for each step addition. Finally, 12 μL of seed solution was

added to the growth solution with gentle up-and-down mixing and the solution were kept undisturbed at 28 ℃ overnight. The color of the solution changed over time depending on the growth of AuNR long axis. The brown color solution is indicative of gold nanorods with width of 12 nm and length of 48 nm. Then, the resulting solutions were centrifuged twice at 5000 rpm for 10 min and were dispersed in deionized water.

Synthesis of Gold Nanocubes (AuNCs). 5mL of 200 mM CTAC and 5mL of 0.5mM $HAuCl_4$ were added to a 50 mL r.b.f. in a water bath at 31 ℃ under magnetic stirring at 500 rpm, and 0.45mL of 20mM ice-cold $NaBH_4$ was added to this solution and mixed for 2min. The resulting dark brownish seed solution left undisturbed at 31℃ for 1 hr, and then, the growth solution was prepared in two 50 mL r.b.f. labeled A and B. 5mL of 200mM CTAC, 4.625mL of deionized water, 250 μL of 10mM $HAuCl_4$, 10 μL of 10mM sodium bromide (NaBr), and 90μL of 40mM L-ascorbic acid were added to both r.b.f.s. Finally, 30 μL of seed solution was added to the growth solution A under magnetic stirring at 80 rpm until the solution color turned light red (~15s). Then, 70 μL of the solution A was transferred to growth solution B under magnetic stirring at 80 rpm for 10 s. Before the solution color change from transparent to pink color, stirring bars in r.b.f. should be removed, followed by keeping undisturbed in 26℃ for more than 15min. The resulting purpled pink color solution is indicative of AuNCs having an edge length of 51.05nm. Then, the solutions were centrifuged twice at 5000 rpm for 10 min and were dispersed in deionized water.

Adsorption Isotherms of Aβ Co-Aggregates with AuNPs. To quantify the amount of adsorbed Aβ peptides onto the surface of AuNPs, FITC labeled amyloid β 1-42 (FITC- Aβ, Bachem AG, Bubendorf, Switzerland) was incubated for 30min with several types of AuNPs. Lyophilized FITC- Aβ was resolved in DMSO at first and the solution was stored at -80 °C as 30 μl of aliquots. This aliquot was diluted with 100 μl of 10mM PB (pH7.4) just before use,

and then, the solution was centrifuged at 11000 rpm for 15 min along with acquiring supernatant only. In order to measure binding affinity of Aβ peptides to AuNPs, 10 μl of AuNPs was mixed with 100 μl of Aβ solution and incubated at room temperature for 30min. Here, the concentration of AuNPs was same with dark-field and TEM experiments and concentration of Aβ peptides was varied from 10 nM to 50 μM. Thereafter, those samples were also centrifuged at 5000 rpm for 1 min except 20-nm AuNPs samples were centrifuged at 8000 rpm for 1 min, and supernatants of samples were collected and fluorescence signals were obtained through Synergy™ MX (BioTek Instruments, Inc, Winooski, VT, USA) at 532 nm.

Size and Zeta Potential of AuNPs with Aβ. Aβ aliquot was diluted with 10mM PB (pH 7.4) for final concentration of 25 μM and centrifuged at 11000 rpm for 15 min. The size and surface charge of AuNPs were measured before and after 30 min co-incubation. In the case of 30 min incubation sample, it was usually centrifuged at 5500 rpm for 1min in order to precipitate only AuNPs enclosed by Aβ peptides except for 20-nm AuNPs which were centrifuged at 8000rpm for 1min. Supernatant was removed and then precipitated AuNPs were dispersed with 10 mM PB (pH 7.4). Then, the size and zeta potential were obtained through Zetasizer (Malvern, Worcestershire, UK).
